# Evaluation of Chemical and Morphological Properties of Spruce Wood Stored in the Natural Environment

**DOI:** 10.3390/polym15244734

**Published:** 2023-12-18

**Authors:** Iveta Čabalová, Michal Bélik, Viera Kučerová, Tereza Jurczyková, Tatiana Bubeníková

**Affiliations:** 1 Department of Chemistry and Chemical Technologies, Faculty of Wood Sciences and Technology, Technical University in Zvolen, T. G. Masaryka 24, 960 01 Zvolen, Slovakia; mbodkabelik@gmail.com (M.B.); viera.kucerova@tuzvo.sk (V.K.); bubenikova@tuzvo.sk (T.B.); 2 Department of Wood Processing, Czech University of Life Sciences in Prague, Kamýcká 1176, 165 21 Prague, Czech Republic; jurczykova@fld.czu.cz

**Keywords:** spruce wood, wood degradation, chemical composition, pH, cellulose polymerization degree, saccharides, fiber properties

## Abstract

This paper focuses on the changes in chemical structure and fiber morphological properties of spruce wood during 15 months of its storage in an open forest woodshed. From the chemical composition, the extractives, cellulose, holocellulose, and lignin content were determined. The pH value was measured on the wood surface using a contact electrode. Acetic and formic acid, saccharides (glucose, xylose, galactose, arabinose and mannose), and polymerization degree (PD) of cellulose were analyzed using the HPLC method. Fiber length and width were determined using a fiber tester analyzer. After 15 months of storage the content of both cellulose (determined by the Seifert method) and lignin did not change; the quantity of hemicelluloses decreased by 13.2%, due to its easier degradation and less stability compared to cellulose; and the pH value dropped by one degree. HPLC analyses showed a total decrease in the cellulose DP of 9.2% and in saccharides of 40.2%, while the largest decreases were recorded in the quantity of arabinose, by 72%, in the quantity of galactose, by 61%, and in the quantity of xylose, by 43%. Organic acids were not detected due to their high volatility during wood storage. The total decrease in average fiber length was 38.2% and in width was 4.8%. An increase in the proportion of shorter fibers, and a decrease in the proportion of longer fibers, was recorded. It can be concluded that fundamental changes occurred in the wood, which could affect the quality of further products (e.g., chips, pulp, paper, particleboards).

## 1. Introduction

Wood is a biodegradable and dimensionally unstable material; it can be degraded quickly and is influenced by many external factors (e.g., UV radiation, thermal effects, dampness, biological agents, and chemicals) [1,2,3,4,5]. Wood degradation is also affected by time and storage conditions [6]. When storing lignocellulosic materials, one has to bear in mind the risks associated with microbial activity of wood-destroying fungi and bacteria [7,8]. The activity of these agents often leads to a partial decrease in the value of wood, although, in some cases, the wood may even be completely destroyed. Changes in the microscopic structure and chemical composition during wood storage are closely related to changes in mechanical and physical properties. [9]. In addition to chemical changes, changes in the color of wood stored in natural conditions can also occur. These color changes are caused by photodegradation. The penetration depth and initiated chemical changes depend on the wavelength of the radiation [10], which implies that the effects of various light spectra are different. When the wood is exposed to direct solar radiation, UV radiation causes serious damage to wood [11]. Seasoning, thermal effects, electromagnetic radiation, and dampness are external factors that can cause changes in the main components (cellulose, hemicellulose, and lignin) and wood extractives, and their content, and thus affect the wood properties (e.g., color) [12].

The main application of wood not related to the food industry is wood pulp production, which can be further used for producing a wide range of products, primarily paper [13]. Approximately 35% of the total number of harvested trees on all continents are used in the paper industry [14]. Nowadays, most of the utilized wood pulp fibers come from tree species, primarily from non-coniferous as well as coniferous species [15,16].

Some paper production plants process spruce wood to make paper. Wood is an anisotropic material and its physical, chemical, and morphological properties depend on many factors. Understanding the morphological and chemical heterogeneity of wood is important for its best use in the paper industry. The content of the basic components (cellulose, hemicellulose, lignin, and extractives) in the wood cells is not uniform and their weight ratio can vary significantly. The chemical composition of wood depends on the morphological structure, species, and tree age [1]. The morphology of the fiber elements is typical for each and every tree species [17]. Fibers of the coniferous species have, according to Retulainen et al. [18], a length of approx. 2.5 to 5.9 mm and a width of 25–50 μm. Research studies carried out so far have shown that fiber characteristics are associated with the tree species, age, or growth conditions [19]. Several studies were focused on the difference between juvenile and mature wood [20], impacts of forest management, e.g., thinning [21], the impact of fertilization and growth speed on the tracheid length [22,23,24], the impact of genotype on the fiber length [25], the correlation between the tracheid length and cross-section properties [26], or the potential impact of climate change [27]. The study by Buksnowitz et al. [19] clearly confirms that the tracheid length (fiber length) of *Picea abies* (L.) Karst. is affected by the tree age, cambium age, and radial distance from the medulla.

When producing wood pulp, it is important to use wood of certain quality, whereas the key point is the wood’s chemical composition, since there is a direct relation with the effectiveness of the pulping process [28]. For the needs of the pulp and paper production industries and for the production of wood pulp, it is necessary for the solution to penetrate completely into the wood chips. The solution penetrates into the wood chips by diffusing the chemicals into water contained in the wood chips. During the pulp production, the wood is exposed to both high temperature and chemical substances that cause delignification of the wood material. The removal of lignin from wood is necessary for the production of certain types of paper. If the chemicals do not sufficiently penetrate the wood chips, the delignification will be uneven, which will be reflected in the quality of the pulp [29]. Therefore, it is necessary to keep in mind the changes in the structure and chemical composition of wood during storage. Storing wood in paper production has certain disadvantages, as well as advantages. The disadvantages include loss in the yield of biomass and wood pulp, and lower lightness and strength. The loss in biomass during storage depends on the climate and storage conditions. A total of 4–15% of biomass is degraded during storage [30]. On the other hand, the advantages include lower content of the extractives and reduced foam forming during washing. According to Allen et al. [31], 12 months of storage causes a decrease in the amount of extractives to 60–80% of the value of green wood. The yields of extractives decrease quickly mainly at the beginning of the storage period during the first 60 days [32]. A decrease in the quantity of these substances in the stored wood reduces the problem of resin in the paper production process [33]. Wood pulp produced from stored wood requires less refining energy [34]. The study of Ramnath et al. [35] indicates that during the storing process, the overall quantity of extractives decreased by 25–44%. At the same time, the occurrence of bacteria and fungi was detected after the storage.

The aim of the present study was to analyze and evaluate the impact of the storage length on the chemical composition and characterization (extractives, cellulose, hemicelluloses, lignin, cellulose polymerization degree, polydispersity, saccharides, organic acids content, and pH value) as well as morphological properties (fiber length and width) of spruce wood, and subsequently to propose the optimum utilization of such wood, mainly for pulp and paper production.

## 2. Experimental

### 2.1. Materials

Norway spruce wood (*Picea abies* L. Karst.) was used for the experiment. The tree was harvested in the central part of Slovakia (Zvolen-Lukové) in September 2021. The age of the tree was 65 years and the height was approx. 25 m. The trunk was cut at a height of 0.5 m above the ground. A 5 m long part of the trunk (diameter approx. 30 cm) was used for the experiment, and was cut into 1 m long pieces. Immediately after harvesting, one piece was sampled for analysis. Other pieces of wood were placed in an open forest woodshed. After 2, 4, 6, 12, and 15 months of storage, one piece was always used for chemical analyses. Based on a visual evaluation of a circular section (taken from the middle of the trunk), samples for analysis were cut so that they did not contain cracks or defects in the wood. Fiber tester analysis was performed immediately after harvesting, and after 6, 12, and 15 months.

Wood samples were conditioned to unify the wood’s moisture content to 12% (±1%). Air conditioning conditions were 6 % relative humidity and temperature of 20 °C. The moisture content and density of all test specimens were then measured according to ISO 13061-1 [36] and ISO 13061-2 standards [37]. Density was measured from the pith to the perimeter. Density, moisture content, and conditions during wood storage are presented in Table 1.

### 2.2. Methods

#### 2.2.1. Chemical Analyses

Samples were disintegrated into sawdust, and fractions from 0.5 mm to 1.0 mm in size were used for the chemical analyses. According to ASTM D1107-21 [38], the extractives (E) were determined in a Soxhlet apparatus with a mixture of absolute ethanol and toluene for analysis (Merck, Darmstadt, Germany) (2:1, *v*:*v*). The duration of extraction was 8 h with 6 siphonings per hour. The lignin (LIG) content was determined according to Sluiter et al. [39], the cellulose (CEL) according to Seifert [40], and the holocellulose (HOLO) according to Wise et al. [41]. Hemicelluloses (HEMI) were calculated as the difference between the holocellulose and cellulose content. Measurements were performed on four replicates per sample. The results were presented as oven-dry wood percentages.

#### 2.2.2. pH Value

The pH measurement was performed on the wood surface using a SenTix Sur glass-handled contact combination electrode and an InoLab7110 pH meter (WTW, Wellheim, Germany). The method was applied in accordance with ISO 6588-1 [42]. The testing is based on measuring the pH value of the surface (in the transverse and longitudinal direction) of the sample moistened with a single drop of distilled water (pH = 6.54) using a planar-combined electrode [43]. Measurements were performed on six replicates.

#### 2.2.3. Chromatographic Analyses

Chemical changes and macromolecular characteristics of cellulose in wood were determined via chromatographic methods. Monosaccharides (D-glucose, D-xylose, D-galactose, L-arabinose, and D-mannose) were determined according to NREL (National Renewable Energy Laboratory) [39] via high-performance liquid chromatography (HPLC).

Conditions: Agilent 1200 chromatograph (Agilent, Santa Clara, CA, USA), HPX-87P column (Bio-Rad, Hercules, CA, USA), refractive index detector (RI); mobile phase: deionized water; flow rate: 0.6 mL/min; injected volume: 50 μL; temperature: 80 °C.

Cellulose molecular weights and polydispersity were analyzed via size exclusion chromatography (SEC) using an Agilent 1200 chromatograph (Agilent, Santa Clara, CA, USA) [44]. Cellulose samples were derived using phenyl isocyanate to obtain cellulose tricarbanilates (CTCs), which were dissolved in tetrahydrofuran prior to size exclusion chromatography (SEC) analysis [45]. The SEC analyses were performed at 35 °C with tetrahydrofuran at a flow rate of 1 mL/min on two PLgel MIXED B columns (10 μm, 7.5 mm × 300 mm), preceded by a PLgel GUARD column (10 μm, 7.5 mm × 50 mm). Two CTC derivatives were prepared for each sample and each derivative was analyzed twice.

The number average molecular weight is the statistical average molecular weight of all the polymer chains in the sample, and is defined by Equation (1) [46]:

(1) $$Mn=1/\sum_{i=1}^{k}Ci/Mi$$

where Mi is the molecular weight of the *i*-th fraction and Ci is the mass fraction of the *i*-th fraction. Mn can be predicted by polymerization mechanisms and is measured by methods that determine the number of molecules in a sample of a given weight. If Mn is quoted for a molecular weight distribution, there are equal numbers of molecules on either side of Mn in the distribution.

The weight average molecular weight is defined by Equation (2) [46]:

(2) $$Mw=\sum_{i=1}^{k}ci*Mi$$

Compared to Mn, Mw takes into account the molecular weight of a chain in determining contributions to the molecular weight average. The more massive the chain, the more the chain contributes to Mw. Mw is determined by methods that are sensitive to the molecular size rather than just their number, such as light scattering techniques.

In addition to Mw and Mn values, the average degree of polymerization (DP) and degree of polydispersity (PD) are used for the macromolecular characterization of cellulose. DP expresses the number of monosaccharide units in the chain and is defined by Equation (3) [46]:

DP = Mw/162(3)

The polydispersity index is used as a measure of the broadness of the molecular weight distribution of a polymer, and is defined by Equation (4):

PD= Mw/Mn(4)

The larger the polydispersity index, the broader the molecular weight.

The quantity of acids (acetic, formic) was determined in the leachate via HPLC using an Agilent 1200 chromatograph (Agilent, Santa Clara, CA, USA).

Conditions: Column: Polymer IEX H form (Watrex, Prague, Czech Republic); mobile phase: 9 mM H_2_SO_4_; flow rate: 0.5 mL/min; detector: UV 210 nm; temperature: 35 °C.

Measurements were performed on four replicates per sample.

#### 2.2.4. Fibers Length and Width

Mixtures (200 mL) of concentrated CH_3_COOH and 30% H_2_O_2_ (1:1, *v*/*v*) were poured onto the wood samples (weight = 10 g and dimensions = 20 mm × 2 mm × 2 mm). Then, the samples were refluxed for 3 h, suction filtered through a sintered glass filter (S1), and washed with distilled water. The fibers’ morphological properties (length and width) were analyzed using an L & W Fiber Tester (Lorenzen & Wettre, Kista, Sweden). Measurements were performed on a single replicate per sample, and the number of fibers within each population of the replicate ranged from 15,482 to 21,128 cells.

## 3. Results and Discussion

### 3.1. Changes in the Chemical Composition of Wood during Its Storage

**Figure 1:**
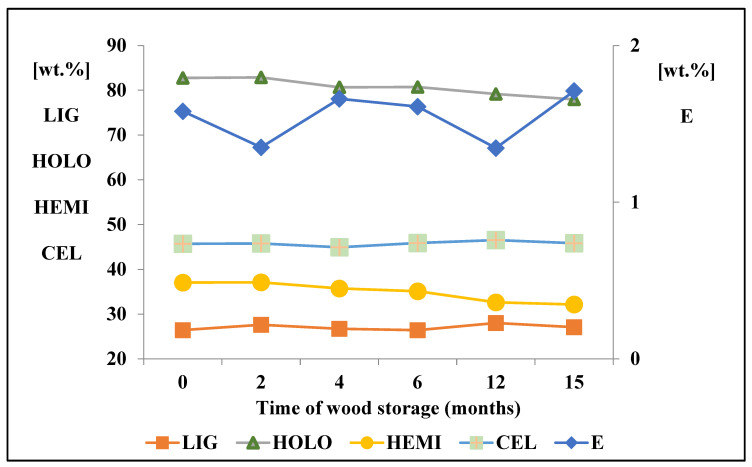
Average chemical composition of spruce wood during 15 months of its storage; LIG—lignin; HOLO—holocellulose, HEMI—hemicelluloses; CEL—cellulose; E—extractives; wt.%—weight percentage of chemical component from wood mass.

This work compared the content of the main chemical components (lignin, hemicelluloses, cellulose, and extractives) of spruce wood, the proportion of which changed with the length of wood storage. The results of the chemical analyses are presented in Figure 1.

The content of extractive compounds was 1.6% at the time of harvesting. After 2 months of storage, the content of extractives decreased by 14%. Bergström and Matison [47] and Freitas et al. [28] describe a decrease in the proportion of extractives by approximately half during the first four weeks of wood storage. According to Holmbom [48], the largest decrease in the quantity of extractives is mainly related to the loss of hydrophilic and also lipophilic substances. Among the chemical components, stilbenes in particular are very sensitive to degradation. In our experiment, after 4 months of storage, we recorded an increase in the quantity of extractives by almost 23% compared to the fresh wood sample. This increase was probably due to the incipient degradation of hemicelluloses. Many authors also describe the increase in the content of extractives during various wood treatments in connection with the degradation of polysaccharides and lignin [49,50,51]. After a year of storage, a decrease in the share of extractives was recorded, which could be related to the storage period (from April to September), when influences such as alternating drought and rain, and high temperature, could be felt on the wood. The increase in the content of extractives after 15 months of storage was probably related to the further degradation of hemicelluloses.

From the main wood chemical components, the changes occurred primarily in the content of hemicelluloses, and the total decrease represented 13.2%. In contrast, the content of cellulose did not change significantly during storage. Several authors described changes in saccharide content in wood during natural aging or thermal treatment. They explain this decrease mainly due to the degradation of the content of hemicelluloses, which are less stable compared to cellulose [52,53,54]. From the main components of wood, hemicelluloses are the most thermally labile components, even at low temperatures, which are normally reached during storage in the natural environment. Compared to cellulose, hemicelluloses have a lower degree of polymerization and their chains are more easily split and relatively easily hydrolyzed under the action of organic acids created as a product of wood degradation [55].

As with cellulose, the content of lignin did not change significantly during wood storage. Similar results are presented in the work of Čabalová et al. [56], where changes in the chemical components of wood during the 8-month storage of spruce wood are described.

Based on the achieved results of chemical analyses and the methods used, it can be concluded that the change in the quantitative chemical composition of the wood stored in a natural environment for 15 months is minimal.

### 3.2. Change in the pH Values of Wood during Its Storage

Acidity is a property of wood that is expressed by means of the pH value. Wood can be more or less acidic in nature and its pH can vary depending on the type of wood, its origin, and other factors. The lower pH value of wood can affect its properties and use. “Acid” wood may have a lower resistance to wood-destroying insects, fungi, molds, or other microorganisms. Low pH can also affect the color and stability of wood. According to Davim [57], the pH of wood ranges from 3.5 to 7.0, while some selected wood species may have a pH outside this range. The pH of spruce wood can also vary depending on various factors, such as the type of soil and the environment in which the tree grows. In general, spruce wood is considered slightly acidic, with a pH ranging from 4.0 to 5.5.

During wood degradation, the following acids can be formed that affect the pH value of wood [58]:

1. Acetic acid, which is produced during the anaerobic fermentation of sugars found in wood. This process is called acetic fermentation and can lead to the formation of not only acetic acid but also ethanol.
2. Formic acid, which is formed in wood through the action of fungi and bacteria. It is a strong acid that can cause metal corrosion.
3. Malic acid, which is formed in wood during its storage as a result of metabolic processes that take place within the wood tissues.

These acids can have a significant effect on the properties of wood and can affect its ability to be used in various applications such as construction, crafts, or even pulp and paper production [58].

An increase or decrease in pH can therefore indicate wood degradation. In our experiment, the pH value was determined using a touch electrode on spruce wood samples that were stored in an outdoor environment (Table 2).

Immediately after harvesting the tree, the wood had an acidic pH in the longitudinal and transverse directions. During storage, the pH value gradually decreased, although in some cases (e.g., after 12 months of storage) it increased compared to the value obtained for fresh wood. This is probably due to the faster evaporation of organic acids from the wood during the summer period. After 15 months of storage, the pH value decreased by one degree compared to the pH of freshly harvested wood. The overall decrease of this characteristic indicates that the wood underwent degradation processes accompanied by the formation of organic acids and influenced by external conditions (temperature, change in humidity, action of microorganisms, etc.). Nurmi et al. [59] analyzed spruce wood, where its pH was 4.5−4.6 at the time of harvesting. However, the pH value increased slightly during storage (3 weeks). pH affects interactions between cellulose chains, which results in changes in the cellulose polymerization degree [60].

### 3.3. Changes in the Content of Organic Acids in Wood during Its Storage

In order to clarify wood degradation, a chromatographic analysis focused on the presence of organic acids, i.e., acetic and formic, was performed in spruce wood stored for 15 months in an outdoor environment. Based on the literature search, there are only a few available scientific works that deal with this topic. They are focused primarily on the creation of emissions of organic acids released from wooden materials in the interior; the formation of organic acids in paper during its aging or during the hydrothermal treatment of wood; and the application of the aforementioned acids to increase anti-fungal protection and wood preservation. Acetic acid is formed in all natural plants, and at a high temperature and relative air humidity, there is an increased formation of acetic acid in wood. Its formation is partly caused by the hydrolysis of esters of acetyl groups in hemicelluloses, which make up approximately one-third of the total carbohydrates in wood [61], but also the side chains of lignin in wood [62]. Hemicellulosic components in deciduous trees comprise approx. 4–6% of the wood weight, and approx. 1–2% in coniferous trees. Therefore, it is generally assumed that softwood produces less acetic acid due to the lower concentration of acetyl esters, but the rate of hydrolysis is also due to the availability of water and the temperature of the environment surrounding the wood. Formic acid production from wood is generally much lower than acetic acid production. Its source of formation is not well understood and one of the theories is the formation of formic acid related to the splitting of pyruvic acid during the metabolic processes of wood [61]. Risholm-Sundman et al. [63] and Ramalho et al. [64] pointed to acetic acid as one of the most widespread substances formed and released from lignocellulosic materials, while acetic acid was proposed as a potential marker for the degradation of lignocellulosic materials [62,65]. The formation of organic acids is observed primarily in museum exhibits made of wood. These acids emitted from wood have a corrosive effect on exposed exhibits [66,67,68]. The study by Barbero-López et al. [69] examines the in vitro antifungal and wood preservative activities of acetic, formic, and propionic acids against the wood decay fungi *Coniophora puteana, Rhodonia* (Poria) *placenta*, *Gloeophyllum trabeum*, and *Trametes versicolor*.

Based on the results presented in Table 3, it can be stated that the content of discussed organic acids was minimal and it was not possible to detect them. However, this does not mean that there are no other components in the wood that reduce the pH of the wood and cause its degradation. The presence of acetic and formic acids was not recorded in the samples stored outdoors. This is explained by their nature as volatile organic acids and their ability to dissolve in water, which could cause them to be easily removed with water.

Hamed et al. [70] analyzed and compared wood stored indoors and outdoors, monitoring the rate of wood degradation using the detection of organic acids. According to their conclusions, the non-air-conditioned indoor environment causes the formation of acids and the influence of this environment on the components of wood is higher than the influence of the outdoor environment, which primarily causes weathering and a significant difference in the rate of acetic acid production between wood types depending on the proportion of acetyl groups, which affect the formation of acetic acid. The rate of acetic acid formation depends on the moisture content of the wood and its temperature. The speed of acid release is also related to the dimensions of the wood. Risholm-Sundman et al. [63] did not report the presence of acetic acid in softwood at room temperature.

A higher acidity of wood was found in the non-air-conditioned indoor environment compared to the outdoor environment. Organic acids emit from wood in moist storage conditions and are active primarily in indoor environments. This action increases at a higher temperature [71]. Therefore, the interaction of forces in the indoor environment can be worse compared to the weather effects. Due to the fact that organic acids were not detected in samples of stored spruce wood, we cannot assess their influence on the rate of degradation of the main chemical components of wood. At this stage, our research did not focus on the detection of other substances that may, due to a reduction in the pH value, have had a degrading effect on the wood’s main components.

### 3.4. Changes in Molecular Weights and Average Degree of Polymerization of Cellulose during Wood Storage

The effect of storage length on changes in macromolecular characteristics of cellulose in spruce wood is presented in Table 4. During 15 months of storage, a total decrease in DP of 9.18% was recorded, which indicates cellulose degradation. We recorded an increase in cellulose DP in the wood taken after 15 months. This was probably caused by the degradation of cellulose shorter chains, which relatively increased the proportion of longer ones. From the results reported by Vojta et al. [72], it is obvious that the greatest decrease in DP of stored spruce wood occurred after 6 months. At the same time, they noted a significant decrease in the mechanical properties of wood. The mechanical properties of the final products are significantly influenced by the macromolecular characteristics of cellulose and its polydispersity [45].

As shown in Table 4, Mn and Mw decreased during storage time. Mn decreased from 14,037 to 12,735 and Mw from 139,542 to 126,622. Polydispersity had a decreasing trend up to 6 months of wood storage, after which the values gradually increased. The formation of acids leads to the shortening of cellulose chains. However, the presence of formic and acetic acid was not detected during our measurement; other acids or substances may have been present that affected the change in pH value and the decrease in DP of cellulose.

Various studies deal with the influence of external conditions on cellulose DP. Xu et al. [73] investigated the effect of temperature and pH on the degradation of cellulose chains of cotton, while clearly confirming the effect of these factors on the changes in cellulose DP. This was caused as an effect of a breakdown of glycosidic bonds in the cellulose molecule, which is reflected in a decrease in cellulose DP. Biotic agents, such as molds and fungi, also have an impact on the decrease in cellulose DP—in some fungi very significantly—which is reflected by a change in the chemical and mechanical properties of wood [74].

### 3.5. Changes in the Content of Saccharides in Wood during Storage

Based on the results of saccharides analysis (Table 5), it can be stated that the changes were mainly manifested in the decrease in the quantity of hemicelluloses.

After 15 months of storage, HEMI decreased by 40.24% compared to freshly harvested wood. Zakzeski et al. [75] investigated the changes in the chemical composition of spruce wood during its storage. They detected a gradual degradation of hemicelluloses in wood after three months of its storage. Even short-term storage has an effect on wood quality, especially on hemicellulose content. Kärki et al. [76] report that wood stored in uncovered piles in the outdoor environment is more easily degraded than covered wood. They describe a significant degradation of hemicelluloses in uncovered piles and an increase in the content of extractives in covered piles.

From the point of view of hemicellulose-type saccharides, there was a degradation of L-arabinose (by 72.0%), D-galactose (by 61.1%), and D-xylose (by 43.0%). Other authors also describe the decrease in the content of L-arabinose in stored wood. Liu et al. [77] monitored the degradation of pine wood (*Pinus radiata*) that was stored for 12 months at the temperatures of 20 °C and 6 °C. They noted a decrease in the content of L-arabinose from 9.6% to 22.4%.

The smallest changes occurred in the content of D-mannose and D-glucose. According to Vidholdová et al. [78], the decrease in the content of saccharides of spruce wood is related to the action of wood fungi. For the further use of wood, it is important to know the degree of degradation of carbohydrates, because, for example, the content of glucomannan influences the strength properties of wood [79].

Pentoses are generally more unstable than hexoses. During our experiments, the degradation of pentoses (D-xylose and L-arabinose) was more intense (a decrease of 50.59%) than the degradation of hexoses (D-glucose, D-galactose, D-mannose), where the decrease was 22.35%. The lower stability of pentoses was also demonstrated during the thermal treatment of wood in the paper by Čabalová et al. [50].

The results in Table 5 show that with a longer time of wood storage, the ratio of saccharides in cellulose to saccharides in hemicelluloses increases. The increase in this ratio is a manifestation of wood degradation and the lower stability of hemicelluloses compared to cellulose, and was also observed in other research works during various thermal treatments of wood [50,80], as well as in naturally aged wood [81,82].

Changes in the content of hemicelluloses have a great influence on the mechanical properties of wood [55,83]. Esteves and Pereira [84] state that the degradation of hemicelluloses is related to their deacetylation, while acetic acid is formed, catalyzing the hydrolysis of glycosidic bonds in polysaccharides and the subsequent reaction of the resulting monosaccharides. Acetic and formic acids were not detected in this work, and therefore their influence on the rate of degradation of hemicellulose-type carbohydrates cannot be assessed by HPLC. However, it has been shown that the pH values decrease, which confirms the degradation of hemicelluloses in any case.

The total decrease in cellulose content was 16.52%. Müller et al. [85] observed changes in the chemical composition of willow and poplar wood during storage. The results showed that there was a decrease of the glucose content by 6–7% and that of glucomannan by 8–9%. They also recorded a reduction in the content of lignin by 4–6%. The decrease in the content of hemicelluloses was most influenced by the length and storage conditions of wood. On the other hand, the content of cellulose was relatively stable. Monitoring changes in the chemical composition of wood chips from poplar and willow wood during 16 weeks of storage was undertaken by Rasmussen et al. [86]. The content of cellulose increased by 11–16% and that of lignin by 6–14%. The above-mentioned authors talk about various factors that affect chemical changes and reduce their content, e.g., microbial activity, enzyme processes, and reactions with oxygen from the air.

Exposure to external influences, and a polluted atmosphere, increases the effect of wood weathering and decomposition of its polysaccharide part. There are gases in the atmosphere that pollute the environment and also diffuse into the internal environment of the wooden material. These are nitrogen oxides, ozone and other photochemical oxidants, sulfur dioxide, and various other particles, internally generated pollutants, formaldehyde, acetaldehyde, and formic or acetic acid [87,88].

In this work, we performed a chemical analysis of the wood, and we found fundamental differences in the used methods. Seifert extraction of cellulose and determination of cellulose tricarbalnylates via Gel Permeation Chromatography (GPC-CTC) are two completely different methods for the determination of cellulose. Seifert cellulose extraction is a process that uses strong acids to dissolve lignin and other non-cellulosic components in wood so that the remaining cellulose fibers can be isolated and measured. This procedure is usually used to obtain cellulose from wood for various industrial purposes. In the case of determining the macromolecular characteristics of cellulose using HPLC, the cellulose is first hydrolyzed to glucose and then the amount of glucose in the solution is measured using HPLC. This method is usually used to measure cellulose in food and medical materials.

After performing the experiments and comparing the results, we found that there are significant differences in determining the content of cellulose. While using the method according to Seifert, we did not detect changes in the amount of cellulose during 15 months of wood storage, but the HPLC method showed a decrease in D-glucose from cellulose by 16.52%. The influence of storage in outdoor conditions and the degradation of cellulose was also confirmed by determining the cellulose DP, while the total decrease in cellulose DP was 9.17%. Differences between the methods were also found in the determination of the content of hemicelluloses. From chemical analysis, we found a total decrease in the amount of hemicellulose after 15 months of wood storage by 13.20%, and via the GPC-CTC method by up to 40.20%.

Since these two methods are intended for completely different purposes, it is not possible to compare them directly, nor to use them on the same samples. Each method has its advantages and disadvantages, and it is necessary to choose the right one for a particular application. It might happen that, within the analysis of one sample, it is possible to obtain different results using these two methods, for example, if the sample contains other components that can affect the extraction of cellulose, such as different types of lignin, volatile organic substances, or different minerals. These ingredients could affect the success of the Seifert extraction and lead to different results. Likewise, different results could be obtained using HPLC if the hydrolysis was not complete. Therefore, it is important to keep in mind that each method has its own limitations and that when comparing results from different methods, it is necessary to take into account all factors that can affect the results. Sroka-Bizoń et al. [89] reached similar conclusions. They compared the Seifert method and HPLC on plant samples. The results showed that the Seifert method gives results with greater inaccuracy than HPLC. HPLC has also shown its advantage in the speed of measurement, and a smaller content of sample is required for measurement. On the other hand, it has a greater financial cost and requires a higher level of measurement expertise. Overall, they found that HPLC is a more suitable method for measuring cellulose in plants than the Seifert method. Antczak et al. [90] compared the efficiency and accuracy of the Seifert and HPLC methods in the determination of cellulose from maple wood (*Acer campestre* L.). Their results showed that the HPLC method had a higher accuracy and efficiency for tent cellulose in wood compared to the Seifert method. In addition, the HPLC method was also faster and easier to use. Due to these advantages, the HPLC method was recommended for the determination of cellulose in wood.

### 3.6. Changes in the Morphological Properties of Wood during 15 Months of Storage

**Figure 2:**
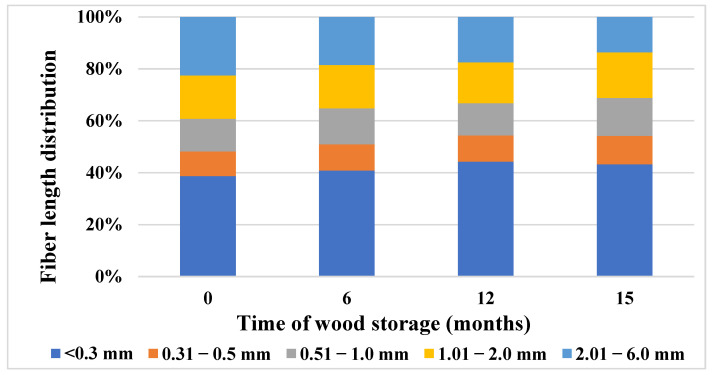
Changes in fiber length distribution during 15-month storage of spruce wood.

**Figure 3:**
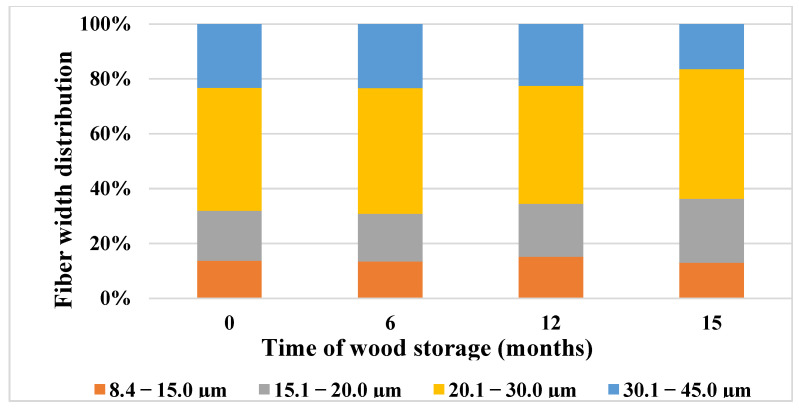
Changes in fiber width distribution during 15-month storage of spruce wood.

From the results of fiber analysis (Figure 2 and Figure 3 and Table 6), it can be stated that there are changes in the morphological properties of stored spruce wood samples. The sample taken from the trunk part of the wood immediately after harvesting contained 38.71% fine fraction (fibers shorter than 0.3 mm). After six months of storage, this share increased by 5.28%, and, after 15 months of storage, by 10.5%. In contrast to the content of the fine fraction, the content of the longest fibers (in the length class from 2.01 to 6.0 mm) decreased during wood storage. Immediately after harvesting, this share represented 22.49%; after 6 months it decreased by 21.3%, and after 15 months, by 65.4%. An increase in the fine fraction and, conversely, a decrease in the fraction of longer fibers, will probably be related to fiber degradation during long-term storage. This is also confirmed in Table 6, which shows the average values of the length and width of the fibers of long-term stored spruce wood. The average fiber length decreased during storage and the overall decrease after 15 months was 38.17% compared to the sample taken immediately after harvesting.

The length of the wood fibers is related to the age, diameter of the tree, position in the trunk, and other factors. The collected samples came only from the stem part (as mentioned in the Methods section). For example, Buksnowitz et al. [19] reported the fiber length of the trunk part of spruce wood up to 7.5 mm, which points to the possibility of its use for specific wood products and for the production of pulp and paper. In our samples, the wood fibers reached a maximum size of 6.7 mm.

Measurement of dimensional characteristics of fibers is also used to assess the rate of wood degradation during various thermal treatments, while, according to Čabalová et al. [91], dimensional characteristics, fiber length, and fiber width decrease with higher heat stress temperature. Sawoszczuk et al. [92] pointed out a decrease in the arithmetic diameter of the fibers, which is linearly correlated with the decrease in the values of the average DP of cellulose.

Based on the results of fiber width analysis (Figure 3), the average width decreased by 4.8% after 15 months of storage (Table 6). At the same time, we noted a decrease in the proportion of fibers in the sample taken after 15 months of storage in the width class from 30.1 μm to 45.0 μm (by 38.42%), and increases in the proportion of fractions in the width classes from 15.1 μm to 20.0 μm (by 28.15%) and from 20.1 μm to 30.0 μm (by 5.54%). This indicates significant changes in fiber width during long-term storage of spruce wood.

Harris [93] and Löönberg et al. [94] reported spruce fiber widths ranging from 29.3 μm to 39.7 μm in grown wood. Based on the data gathered by us, the width of the fibers of the spruce stem ranged from 8.4 μm to 68.0 μm. The average width of spruce fibers in the work published by Gerendiain et al. [95] is 28.7 μm. According to our results, the average width of the fibers was 24.36 µm immediately after harvesting and 23.19 µm after 15 months of storage.

## 4. Conclusions

This research provides useful information about fundamental changes in the chemical structure of wood, fiber morphology, and basic properties of raw material stored over a long period in an outdoor natural environment. Some companies store wood in pieces even for a year. Paper mills normally store raw material in the form of chips for several weeks, depending on the season and the type of raw material. Long-stored wood can be used for pulp production, for particleboard processing, in the building industry, etc. The changes in the anatomical and chemical structures influence all of the properties of particleboards [96] and the quality of pulp [97]. Therefore, it is necessary to handle the findings from this experiment well.

Based on the results, we reached the following conclusions:

- The quantity of extractives decreased by 14% after 2 months of storage. During storage, there was also observed an increase in the content of extractives due to the degradation of other wood components, primarily hemicelluloses.
- The content of cellulose, determined according to Seifert, and the content of lignin, changed minimally.
- Chemical analysis showed a decrease in the content of hemicelluloses by 13.21%, due to its easier degradation and less stability compared to cellulose.
- The pH value after 15 months of wood storage decreased by one degree compared to the pH of freshly harvested wood.
- Organic acids, mainly acetic and formic, were not detected in samples of spruce wood stored in outdoor conditions due to its high volatility.
- After 15 months of storage, a decrease in the cellulose DP by 9.18% was observed, which points to the degradation of cellulose due to the influence of various factors (e.g., humidity, temperature, microorganisms) in the external environment.
- Using the HPLC method, we found a decrease in the quantity of hemicelluloses after 15 months of wood storage, of 40.24%, compared to freshly harvested wood. From the point of view of hemicellulose-type saccharides, the greatest degradation occurred in the content of L-arabinose (by 71.99%), D-galactose (by 61.11%), and D-xylose (by 43.04%). The total decrease in the amount of D-glucose from cellulose was 16.52%.
- A sample taken immediately after harvesting contained a fine fiber fraction of 38.71%; after 6 months of storage this fraction increased by 5.28% and after 15 months of storage this increase was 10.5%.
- The fraction of fibers in the length class from 2.01 to 6.0 mm decreased during storage. Immediately after harvesting this share was 22.49%; after 6 months it decreased by 21.3% and after 15 months it decreased by 65.4%. The average fiber length decreased by 38.17% after 15 months of wood storage.
- The analysis of the fiber width showed that there was a decrease after 15 months of storage, by 4.8%. After 15 months of wood storage, we noted a decrease in the content of fibers in the width class from 30.1 to 45.0 μm, by 38.42%.
- There are some differences in the methods used—the determination of cellulose according to Seifert gave us different data compared to the determination of D-glucose via the HPLC method.
- Several changes in the chemical structure and morphology of the fibers were detected during 15 months of wood storage in an open forest warehouse. However, the quality of the raw material remains very good and we do not expect it to significantly affect the quality of such products as pulp, paper, or composite boards.

## Figures and Tables

**Table 1:** Spruce wood storage conditions, moisture, and density of samples.

Storage Time/ Conditions	Average Air Humidity (%)	Average Air Temperature (°C)	Average Precipitation (mm)	Sample Moisture (%)	Sample Density (g·cm^−3^)
September–harvesting time	-	-	-	9.35	0.420
					(pith: 0.388, perimeter: 0.452)
from September to November	88.22	8.63	0.87	9.29	0.423
	(max. 99.17, min. 69.09)	(max. 19.57, min. −1.52)	(max. 29.6, min. 0.87)		(pith: 0.403, perimeter: 0.448)
from November to January	91.34	−0.47	0.96	6.73	0.448
	(max. 99.17, min. 65.31)	(max. 11.334, min. −7.38)	(max. 15, min. 0)		(pith: 0.426, perimeter: 0.469)
from January to March	72.12	3.45	0.94	6.82	0.443
	(max. 99.17, min. 38.13)	(max. 11.33, min −2.31)	(max. 15, min. 0)		(pith: 0.413, perimeter: 0.466)
from March to September	71.68	17.30	1.59	7.79	0.430
	(max. 99.17, min. 43.25)	(max. 27.80, min. 1.47)	(max. 34.40, min. 0)		(pith: 0.396, perimeter: 0.464)
from September to December	90.89	10.02	1.54	8.64	0.441
	(max. 100, min. 69.23)	(max. 19.46, min −0.28)	(max. 18.2, min 0)		(pith: 0.423, perimeter: 0.459)

**Table 2:** Changes in the pH values of spruce wood during 15 months of its storage. Data are presented as mean ± standard deviation (SD).

Time of Wood Storage (Months)	pH Values	
	Transverse Direction	Longitudinal Direction
0	5.32 ± 0.29	5.26 ± 0.22
2	4.25 ± 0.22	5.19 ± 0.17
4	4.43 ± 0.08	4.58 ± 0.26
6	4.59 ± 0.37	4.17 ± 0.06
12	4.89 ± 0.31	4.49 ± 0.34
15	4.28 ± 0.28	4.23 ± 0.58

**Table 3:** Content of acetic and formic acids in spruce wood during 15 months of its storage.

Time of Wood Storage (Months)/ Amount of Organic Acids	0	2	4	6	12	15
acetic acid (mL/L)	Ui	Ui	Ui	Ui	Ui	Ui
formic acid (mL/L)	Ui	Ui	Ui	Ui	Ui	Ui

Ui—unidentified.

**Table 4:** Changes in macromolecular characteristics of cellulose during 15 months of wood storage.

Time of Wood Storage (Months)/ Characteristics	0	2	4	6	12	15
Mn	14,037 ± 222	13,740 ± 48	13,550 ± 56	13,577 ± 29	12,858 ± 30	12,735 ± 47
Mw	139,542 ± 2142	136,971 ± 700	119,874 ± 741	120,197 ± 589	121,024 ± 170	126,622 ± 387
PD	9.94 ± 0.03	9.97 ± 0.02	8.85 ± 0.02	8.86 ± 0.02	9.41 ± 0.01	9.94 ± 0.01
DP	861 ± 13	846 ± 4	740 ± 5	742 ± 4	747 ± 1	782 ± 2

Mn—the number average molecular weight; Mw—the weight average molecular weight; PD—polydispersity; DP—cellulose polymerization degree; Data are presented as mean ± standard deviation (SD).

**Table 5:** Changes in the content of saccharides presented in spruce wood during 15 months of its storage.

Time of Wood Storage (Months)/ Saccharide Content (mg/g)	0	2	4	6	12	15	a Total Decrease (%)
GLC	460.7 ± 3.5	470.1 ± 2.4	466.1 ± 2.0	479.1 ± 0.5	517.9 ± 0.9	379.9 ± 1.8	17.5
XYL	59.4 ± 1.5	60.7 ± 1.2	61.3 ± 1.1	65.2 ± 1.0	66.7 ± 1.2	33.8 ± 0.4	43.0
GAL	37.5 ± 0.7	37.0 ± 1.1	33.9 ± 1.5	39.12 ± 1.0	17.21 ± 0.8	14.6 ± 0.6	61.1
ARA	21.0 ± 1.8	13.0 ± 0.4	16.2 ± 0.5	13.9 ± 0.7	7.6 ± 0.2	5.9 ± 0.1	72.0
MAN	110.3 ± 3.0	110.7 ± 2.0	11.6 ± 0.2	102.3 ± 1.1	110.0 ± 3.4	78.0 ± 0.5	29.3
SUM SACCH	688.9 ± 6.0	691.5 ± 2.2	689.2 ± 3.0	699.6 ± 1.4	719.5 ± 1.3	512.2 ± 0.8	26.6
GLC from HEMI	36.8 ± 1.0	36.9 ± 0.7	37.2 ± 0.1	34.1 ± 0.4	36.7 ± 1.1	26.1 ± 0.2	29.3
GLC from CEL	423.9 ± 3.7	433.2 ± 3.0	428.9 ± 2.0	445.0 ± 0.3	481.2 ± 1.8	353.9 ± 2.0	16.5
SUM HEMI	264.9 ± 4.1	258.3 ± 2.0	260.3 ± 1.1	254.6 ± 1.6	238.3 ± 2.7	158.3 ± 1.5	40.2
SUM PENT	80.3 ± 2.0	73.7 ± 1.5	77.6 ± 0.8	79.1 ± 0.8	74.4 ± 0.8	39.7 ± 0.4	50.6
SUM HEX	608.5 ± 4.4	617.8 ± 2.3	611.6 ± 3.4	620.5 ± 1.2	645.1 ± 1.2	472.5 ± 0.7	22.4
Ratio CEL/HEMI	1.60	1.68	1.65	1.75	2.02	2.24	-

GLC—D-glucose; XYL—D-xylose; GAL—D-galactose; ARA—L-arabinose; MAN—D-mannose; SUM SACCH—sum of saccharides; GLC z HEMI—D-glucose from hemicelluloses; GCL z CEL—D-glucose from cellulose; SUM PENT—sum of pentoses (XYL + ARA); SUM HEX—sum of hexoses (GLC + GAL + MAN); Data are presented as mean ± standard deviation (SD).

**Table 6:** Changes in the average fiber length and width during 15 months of wood storage.

Time of Wood Storage (Months)	0	6	12	15
average fiber length (mm)	1.31	1.15	0.88	0.81
average fiber width (µm)	24.36	24.19	23.80	23.19

## Data Availability

Data are contained within the article.
